# Supplementary material for: Honeybees Use Multiple Invariants to Control Their Altitude
Source: Insects. 2023 Mar 24;14(4):313. doi: 10.3390/insects14040313 (PMC10146580; doi:10.3390/insects14040313)
Supplement: Supplementary file 1 [file insects-14-00313-s001.zip › insects-2135143-supplementary.pdf]

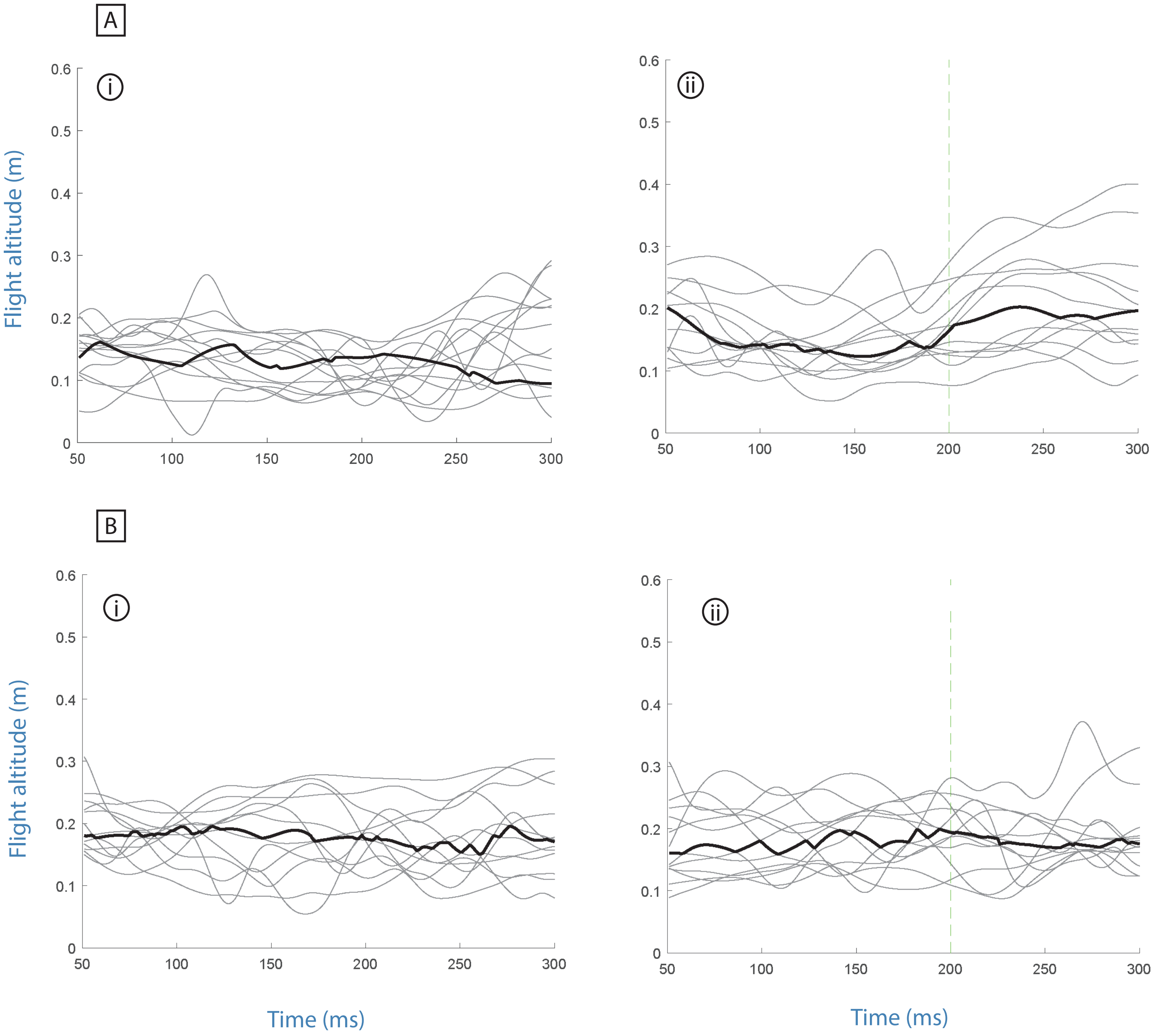

Figure. S1 - Individual time course of altitude changes in two experimental conditions of experiment 1: A.i and B.i are control conditions, A.ii is the dynamic converging condition and B.ii is the dynamic diverging condition above white ground. While the altitude remains unchanged in both the control and diverging conditions, an increase in altitude can be observed after the perturbation (green dotted line) in converging condition. 13 randomly selected flights are plotted in each condition, each one respecting a ground-following criteria (see section 5.A). Median altitude is represented in bold. The data was acquired at 100Hz, then low-pass filtered by a second-order Butterworth filter with a cut-off frequency of 3Hz to smooth trajectories. Corresponding boxpots are visible in Fig.6-A,B.

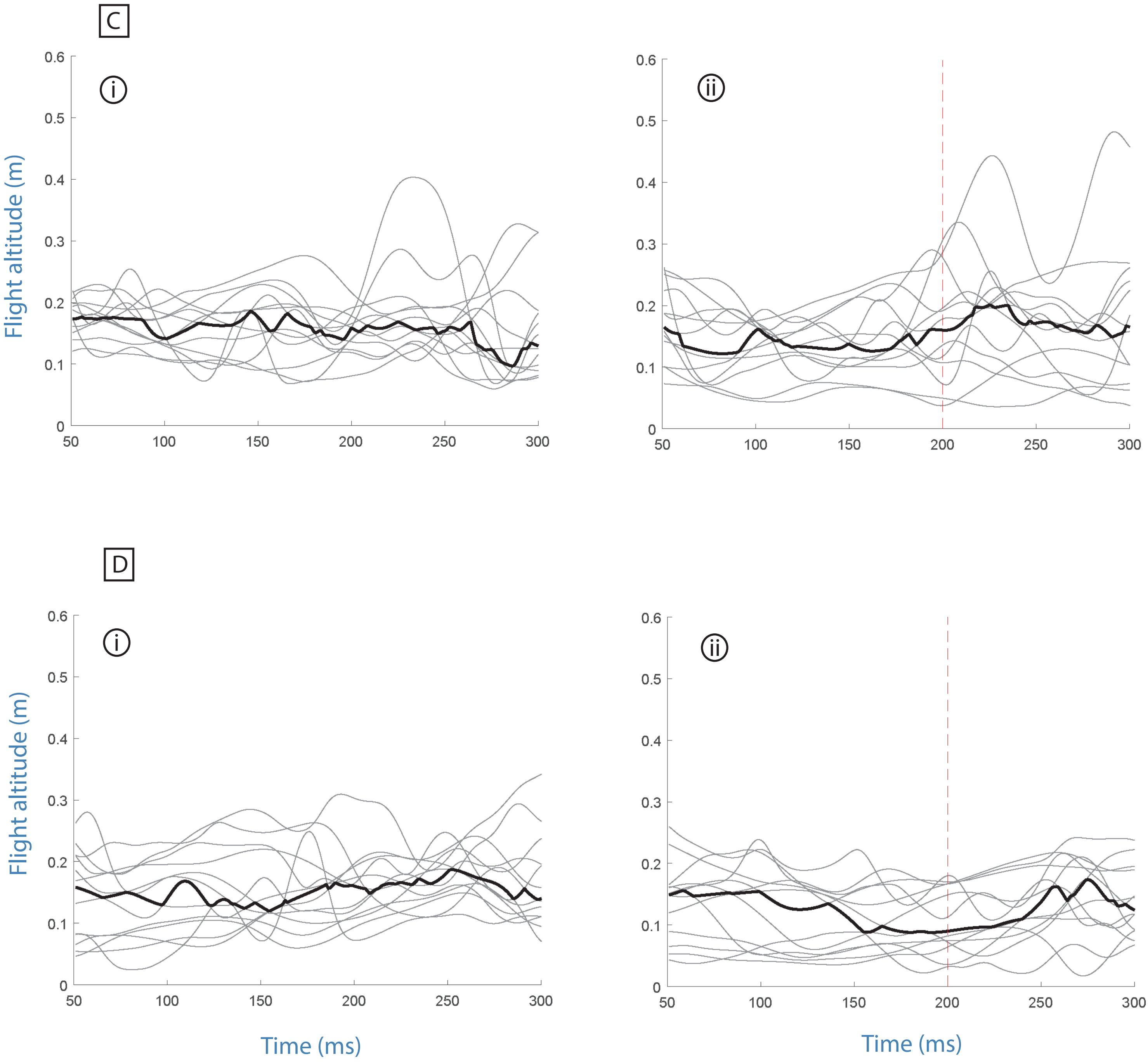

Figure. S2 - Individual time course of altitude changes in four experimental conditions of experiment 1: C.i and D.i are control conditions, C.ii is the dynamic converging condition and D.ii is the dynamic diverging condition above striped ground. The altitude remains unchanged in every condition. Randomly selected flights are plotted in each condition, each one respecting a ground-following criteria (see section 5.A). Median altitude is represented in bold. The data was acquired at 100Hz, then low-pass filtered by a second-order Butterworth filter with a cut-off frequency of 3Hz to smooth trajectories. Corresponding boxpots are visible in Fig.6-C,D.

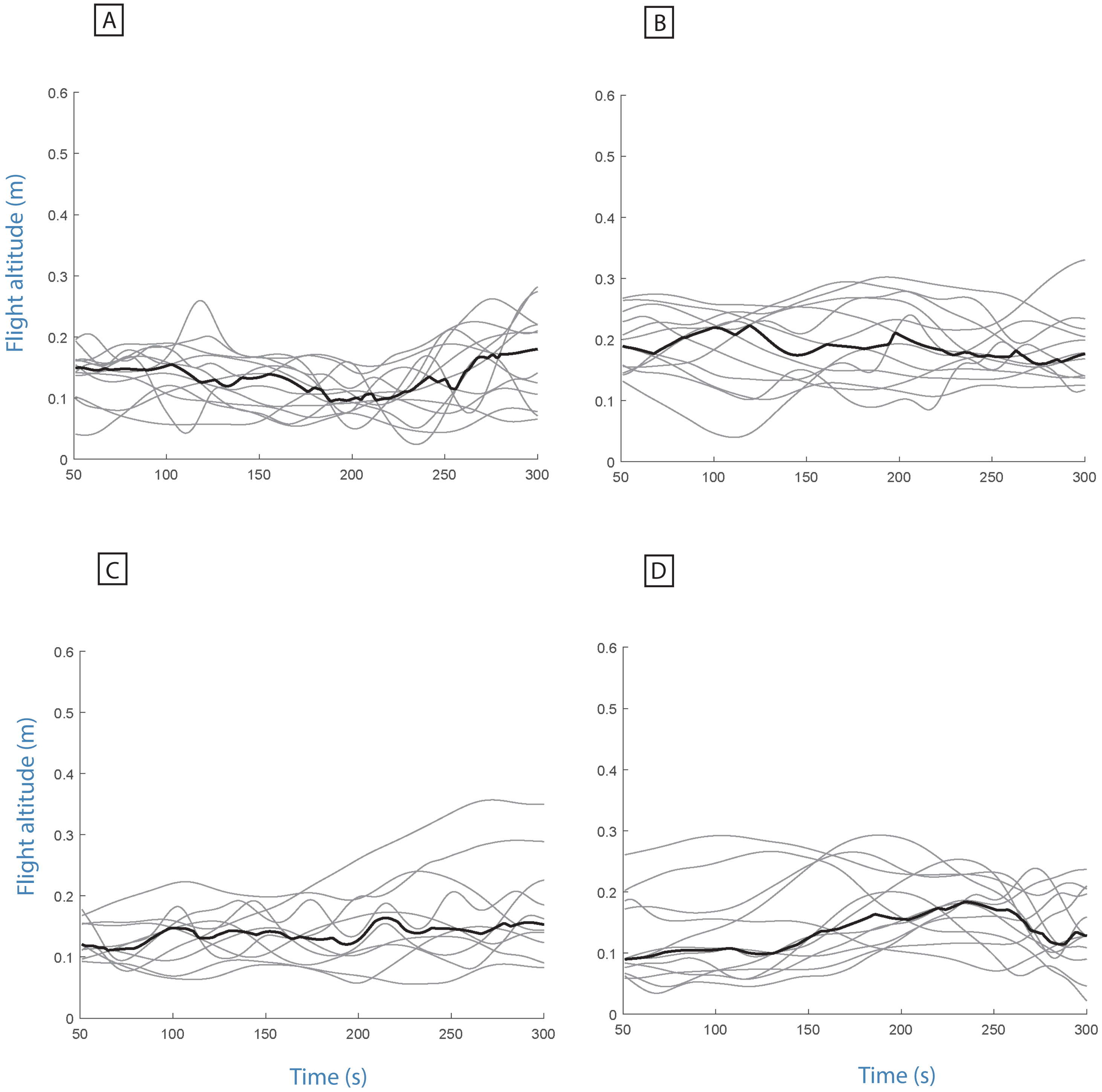

Figure S3 - Individual time course of altitude changes in four experimental conditions of experiment 2: A is the static converging condition above white ground, B is the static diverging condition above white ground, C is the static converging condition above striped ground and D is the static diverging condition above striped ground. The altitude remains unchainged in every condition. Randomly selected flights are plotted in each condition, each one respecting a ground-following criteria (see section 5.A). Median altitude is represented in bold. The data was acquired at 100Hz, then low-pass filtered by a second-order Butterworth filter with a cut-off frequency of 3Hz to smooth trajectories. Corresponding boxpots are visible in Fig.7. Corresponding control conditions are visibe in Fig.S1-A.i,B.i and Fig.S2-C.i,D.i.
